# Different autonomic responses to occupational and leisure time physical activities among blue-collar workers

**DOI:** 10.1007/s00420-017-1279-y

**Published:** 2017-11-24

**Authors:** Tatiana O. Sato, David M. Hallman, Jesper Kristiansen, Jørgen H. Skotte, Andreas Holtermann

**Affiliations:** 1 Physical Therapy Department, Federal University of São Carlos (UFSCar), Rodovia Washington Luís km 235, São Carlos, SP 13565-905 Brazil; 2 Department of Occupational and Public Health Sciences, Centre for Musculoskeletal Research, University of Gävle, 801-76 Gävle, Sweden; 3 National Research Centre for the Working Environment (NRCWE), Lersø Parkallé 105, 2100 Copenhagen Ø, Denmark; 4 Institute of Sports Science and Clinical Biomechanics, University of Southern Denmark, Odense, Denmark

**Keywords:** Heart rate variability, Cardiovascular disease, Objective measurements, Occupational health

## Abstract

**Purpose:**

The differential effect of occupational and leisure time physical activity on cardiovascular health is termed the physical activity health paradox. Cardiac autonomic modulation could bring insights about the underlying mechanism behind this differential effect. The aim was to compare heart rate variability (HRV) during different activities (sitting, standing and moving) at work and leisure among blue-collar workers.

**Methods:**

One hundred thirty-eight workers from the NOMAD cohort were included. Data from physical activity and HRV were obtained for 3–4 days using tri-axial accelerometers (Actigraph GT3X+) and a heart rate monitor (Actiheart). HRV indices were determined during sitting, standing and moving both at work and leisure. Linear mixed-models with two fixed factors (activities and domains) were applied to investigate differences in HRV indices adjusting for individual and occupational factors.

**Results:**

The results showed significant effects of domain (*p* < 0.01), physical activity type (*p* < 0.01) and interaction between domain and activity type (*p* < 0.01) on HRV indices. Mean heart rate (IBI) and parasympathetic measures of HRV (RMSSD and HF) were lower for sitting (*p* < 0.01) and higher for moving (*p* < 0.01) during work compared with leisure, while no difference between domains was found for standing (*p* > 0.05). Sympathovagal balance (LF/HF) was higher during work for sitting and moving (*p* < 0.01), but showed no difference for standing (*p* = 0.62).

**Conclusions:**

Differences in cardiac autonomic modulation between work and leisure were found, indicating sympathetic predominance during work and parasympathetic predominance during leisure for sitting. Autonomic responses can be part of the mechanism that explains the differential effect of occupational and leisure time physical activity on health.

## Introduction

It is well-established that high physical activity at leisure time decreases risk for all cause and ischemic heart disease mortality (Pedersen and Saltin 2015; Warburton and Bredin 2016). On the other hand, high physical activities at work showed an increased risk for the same mortality indicators (Holtermann et al. 2009, 2010). Thus, it seems to be an inverse relationship between occupational (OPA) and leisure time physical activity (LTPA) and cardiovascular risk (Li et al. 2013; Krause et al. 2015), termed the physical activity health paradox (Holtermann et al. 2012a). The underlying mechanisms for the physical activity health paradox remain unknown. However, it may be related to different autonomic responses during physical activity (Hallman et al. 2017).

Heart rate variability (HRV) analyses can be used as a reliable indicator of autonomic regulation in response to different daily activities, both in the occupational context as well as during leisure time (Guijt et al. 2007; McNarry and Lewis 2012; Hallman et al. 2015a).

Autonomic nervous system modulation is intrinsically related to physical activity via the sympathetic and parasympathetic nervous systems (Pomeranz et al. 1985; Bernardi et al. 1996; Chan et al. 2007; Billman 2011). Thus, comparing HRV for the same physical activity types during work and leisure time might help understanding if work has a differential effect on autonomic activity compared to leisure. However, due to the dependence of HRV indices on body posture and activity (Bernardi et al. 1996; Perini and Veicsteinas 2003; Rennie et al. 2003; Chan et al. 2007; Watanabe et al. 2007) it is important to compare the HRV indices during the same physical activity types and postures during work and leisure.

The aim of this study was to determine whether HRV measured during different activity types, such as sitting, standing and moving, differs between work and leisure in blue-collar workers. These results might bring further insight about the potential mechanisms behind the physical activity health paradox connections between OPA and LTPA and autonomic modulation and health.

## Methods

### Study population and exclusion criteria

This study is based on data from the cross-sectional study called “New method for Objective Measurements of physical Activity in Daily Life (NOMAD)”, conducted on blue-collar workers recruited from seven workplaces in Denmark. To be included in the study the subjects must have the possibility to participate during paid working time, to be employed for more than 20 h per week and being between 18 and 65 years. Exclusion criteria were declining to sign the informed consent, pregnancy, diabetes, cardiovascular diseases, medication prescription, and fever on the testing day. Allergy to band aid caused exclusion from the objective measurements. Population and recruitment were described in detail elsewhere (Gupta et al. 2015; Hallman et al. 2015b).

Data were obtained from 237 blue-collar workers. Subjects not filling out the questionnaire and those not wearing the objective measurements (*n* = 77) were excluded. Additionally, workers with less than 7 h (i.e. at least 7 day) of valid HRV recordings, for both work and leisure time were excluded (*n* = 22). Thus, the sample was composed by 138 blue-collar workers. The main occupational groups were manufacturing laborers (*n* = 38); assemblers (*n* = 27); mining and construction laborers (*n* = 24); cleaners (*n* = 23); personal care workers in health services (*n* = 12); garbage collectors (*n* = 9); heavy truck drivers and mobile plant operators (*n* = 4) and other elementary workers (*n* = 1).

All subjects were informed about the study prior to participation and provided an informed consent. The study was approved by the local ethics committee (Journal number H-2-2011-047) and was conducted in accordance with Helsinki declaration.

### Procedure

#### Assessment of individual and occupational factors

A self-reported digital questionnaire was administered to the workers including age (discrete variable; in years), gender (dichotomous variable; female or male), tobacco use (dichotomous variable; yes or no), physical activity at leisure time (categorical variable; almost completely physically passive < 2 h/week, light physically active 2–4 h/week, physically active for 2–4 h/week, more strenuous physical activity > 4 h/week) and lifetime occurrence of medical diagnoses of hypertension, depression or other mental diseases (dichotomous variable; yes or no).

Occupational factors were also collected by the questionnaire, and included job seniority (discrete variable; in months), lifting and carrying during work (categorical variable; almost all the time, approximately 3/4 of the time, approximately 1/2 of the time, approximately 1/4 of the time, rarely/very little or never) and influence at work (discrete variable 0–100%). Influence at work was measured using four items from the Copenhagen Psychosocial Questionnaire (Pejtersen et al. 2010) and a higher number indicates more influence at work.

Height (cm) was measured using a scale (Seca, model 123) and weight (kg) was measured by a digital scale (Tanita modelo BC 418 MA). Body mass index (BMI) was calculated according to the formulae BMI = weight (kg)/height^2^ (m). Subjects also performed a submaximal fitness test on a cycle ergometer to obtain their aerobic capacity (Astrand 1960).

#### Assessment of the activities and HRV

Workers were asked to wear the devices, i.e. the accelerometers and the Actiheart sensors, for four continuous days, ideally a period covering two working days, and one-two days off work. The workers were instructed to not remove the equipments, including while bathing and sleeping, unless in case of itching or any kind of discomfort.

Physical activities were objectively recorded using accelerometers (ActiGraph GT3X+, Actigraph, Florida, USA), which measured the acceleration in three dimensions at 30 Hz with a range of 6G (1G = 9.81 m/s^2^). The accelerometers were attached to the hip (laterally and below the right iliac crest) and thigh (medial on the right thigh), mounted with the x-axis pointing downwards (up/down), and y-axis and z-axis oriented horizontally (Skotte et al. 2014). An activity diary was also provided to the worker to obtain data about the time of the following events: get up in the morning, start and finish work, bedtime and time of reference measurement.

The files were initialized for recording and downloaded using the manufacturer’s software (ActiLife, version 5.5) and afterwards the Acti4 software (The National Research Centre for the Working Environment, Copenhagen, Denmark and BAuA, Berlin, Germany) was used to detect activity types: sitting, standing still and moving (slow and fast walking, running, walking stairs, cycling). This software detected each activity with high sensitivity and specificity, allowing for precise and valid identification of activities. Details of the activities definition have been published elsewhere (Skotte et al. 2014; Stemland et al. 2015).

HRV was derived from the Actiheart system (Camntech Ltd, Cambridge, UK), which measures electrocardiography with a sensitivity of 0.250 mV. The sensor was attached below the apex of the sternum and the horizontal wire was fixed at the right side at the level of the 5th and 6th intercostal space. Respiratory rate was not controlled during data collection. Data were sampled at 128 Hz and it was processed using a band-pass filter (10–35 Hz). The power spectrum was obtained through the robust period detection method. Details of the heart rate variability data processing have been published elsewhere (Kristiansen et al. 2011; Skotte and Kristiansen 2014).

Based on the RR intervals series, HRV was analyzed from 5-min windows with less than 10% erroneous inter beat intervals (IBI), both in the time and frequency domains. Abnormal beats were automated removed before analyzing HRV. The time domain HRV indices were mean IBI (ms), RMSSD (square root of the mean squared differences of successive IBI) and SDNN (standard deviation of IBI). In the frequency domain of HRV, spectral power density was calculated in the low (LF 0.04–0.15 Hz) and high frequency (HF 0.15–0.4 Hz). Mean IBI and SDNN are measures of the mean heart rate and heart rate variability, respectively. RMSSD and HF are indicators of the parasympathetic modulation of cardiac rhythm (Malik et al., 1996; Michael et al. 2017), while LF is taken as an indicator of sympathetic modulation of cardiac rhythm although it is recognized that parasympathetic modulation also contributes to LF (Malik et al. 1996; Michael et al. 2017). The sympathovagal balance (LF/HF) was also calculated (Malik et al. 1996; Kristiansen et al. 2011; Skotte and Kristiansen 2014).

### Statistical analyses

All HRV variables, except IBI had non-normal distributions according to the Kolmogorov Smirnov test (*p* < 0.05). Thus, non-normally distributed variables were transformed using the natural logarithm (ln) prior to further analyses.

Linear mixed-models with two fixed factors (activity type, 3-levels × domains, 2-levels) were applied to investigate differences in the HRV indices between activity types (sit, stand and move), domains (work and leisure) and their interaction. The covariance type was unstructured and the restricted maximum likelihood (REML) estimation method was chosen. Pairwise comparisons were done as a post hoc test using the estimated marginal means. Unadjusted and fully adjusted models were estimated. For the adjusted model the covariates age, sex, BMI, smoking, physical activity at leisure time, job seniority, lifting and carrying and influence at work were included as fixed effects. Subject and intercept were included as random effects. Stratified analyses on smoking (yes or no) and influence at work (high = above or equal the median value of 65, or low = below the median value of 65) were also performed. All statistical analyses were performed using SPSS software (version 17.0) and the level of significance was set at 5%.

## Results

One hundred thirty-eight blue-collar workers were included in the statistical analyses and the main characteristics of the workers are presented in Table 1. The mean age of the workers was 45.2 years. Out of the 138 workers, 51.4% were females, 42.0% were smokers, 18.0% reported lifetime occurrence of hypertension and 45.2% reported to perform lifting and carrying for more than half of the work time. The mean (SD) number of measured days was 2 (0.9), with a minimum of 1 and a maximum of 4 days. The mean (SD) of valid accelerometer wear time per day was 8.6 (2.3) h for work and 8.5 (2.5) h for leisure time. The mean (SD) of valid Actiheart wear time per day was 10.7 (5.5) h for work and 16.3 (12.9) h for leisure time. Time spent sitting was higher during leisure time and time spent in standing still and moving were higher during work time.

**Table 1:** Individual and occupational characteristics among 138 blue-collar workers in the NOMAD cohort

	*n* (%)	Mean (SD)	Minimum–maximum
Females	71 (51.4)		
Age (years)	138	45.2 (9.8)	25–65
Body mass index (kg/m^2^)	138	25.8 (4.7)	17.4–40.7
Smokers	58 (42.0)		
Life-time occurrence of medical diagnoses			
Hypertension	25 (18.1)		
Depression or other mental diseases	20 (14.5)		
Aerobic capacity^a^ (mlO_2_/min/kg)	106	32.9 (8.2)	15.7–56.5
Low	69 (65.1)		
Medium	23 (21.7)		
High	14 (13.2)		
Objectively measured MVPA during leisure time (h/day)	138	0.6 (0.4)	0.1–2.2
Seniority in the current occupation (months)	131	165.9 (141.3)	1–576
Lifting and carrying during work	137		
Almost all the time	7 (5.1)		
Approximately 3/4 of the time	26 (19.0)		
Approximately 1/2 of the time	29 (21.2)		
Approximately 1/4 of the time	31 (22.6)		
Rarely/very little	38 (27.7)		
Never	6 (4.4)		
Influence at work (scale 0–100)	136	54.5 (17.4)	20–100
Time sitting (h/day)			
Work	138	3.1 (1.5)	0.5–6.6
Leisure	138	5.5 (1.8)	2.0–11.9
Time standing (h/day)			
Work	138	2.4 (1.3)	0.3–5.2
Leisure	138	1.5 (0.8)	0.3–4.8
Time moving (h/day)			
Work	138	1.7 (0.9)	0.3–3.8
Leisure	138	0.8 (0.5)	0.2–2.8

^a^Classification based on age and gender according to the Danish Heart Association

*NOMAD* new method for objective measurements of physical activity in daily life, *MVPA* moderate to vigorous physical activity

**Fig. 1:**
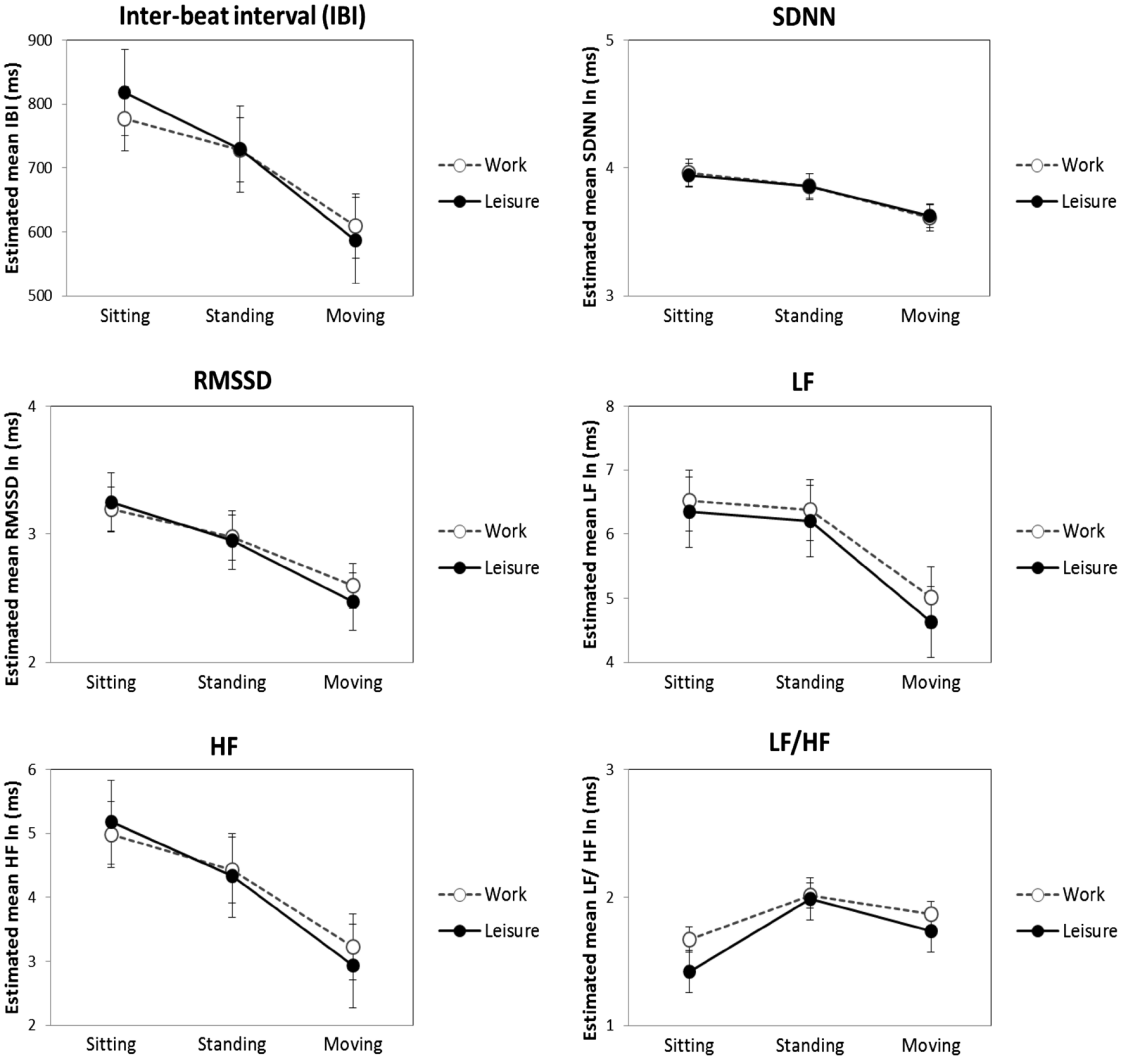
Estimated mean values and confidence intervals for heart rate variability indices during sitting, standing and moving at work and leisure domains according to the fully adjusted model

The mean and standard deviation for the HRV indices obtained during work and leisure domains for each activity type are presented in Table 2 and the results from the crude and fully adjusted linear mixed models are shown in Table 3. Considering the adjusted model, the main effect of domain was only significant for LF and LF/HF (*p* < 0.01). That is, sympathetic-related measures of HRV and sympathovagal balance were higher during work than during leisure. The main effect of activity was significant for all HRV indices (*p* < 0.01), with higher estimates for sitting and standing in relation to moving, except for sympathovagal balance (LF/HF). Compared to the main effect of domain, the estimates for the activities were larger, indicating that the effect of activity is more pronounced than the effect of domain for all HRV indices. The interaction between domain and activity type was significant for IBI, RMSSD, HF and LF/HF (*p* < 0.01) in the adjusted model (Fig. 1). According to this model, mean heart rate (IBI) and parasympathetic measures of HRV (RMSSD and HF) were lower for sitting (*p* < 0.01) and higher for moving (*p* < 0.01) during work compared with leisure time, while no difference between domains was found for standing (*p* > 0.05). On the other hand, sympathovagal balance (LF/HF) was higher during work for sitting and moving (*p* < 0.01), but showed no difference for standing (*p* = 0.62).

**Table 2:** Heart rate variability indices during work and leisure time stratified on physical activity type among blue-collar workers in the NOMAD cohort. Data are presented as mean (SD)

Variables	Sitting		Standing		Moving	
	Work (*n* = 138)	Leisure (*n* = 138)	Work (*n* = 117)	Leisure (*n* = 126)	Work (*n* = 118)	Leisure (*n* = 98)
IBI (ms)	773.7 (92.8)	815.2 (103.9)	727.6 (87.0)	728.3 (103.6)	611.1 (77.5)	586.8 (84.0)
SDNN (ms)	55.9 (19.6)	54.5 (17.6)	51.5 (19.7)	50.8 (18.1)	39.0 (12.3)	39.9 (13.8)
ln SDNN	3.97 (0.34)	3.95 (0.32)	3.87 (0.38)	3.87 (0.35)	3.62 (0.29)	3.63 (0.35)
RMSSD (ms)	27.3 (13.2)	28.5 (12.9)	22.4 (10.8)	21.4 (10.5)	14.4 (5.5)	13.1 (6.5)
ln RMSSD	3.21 (0.45)	3.26 (0.43)	3.01 (0.44)	2.96 (0.46)	2.60 (0.39)	2.46 (0.47)
LF (ms^2^/Hz)	920.8 (715.5)	765.5 (575.6)	931.6 (883.8)	740.6 (670.8)	212.6 (157.8)	202.5 (299.2)
ln LF	6.54 (0.78)	6.37 (0.77)	6.44 (0.92)	6.24 (0.90)	5.05 (0.85)	4.57 (1.28)
HF (ms^2^/Hz)	246.0 (313.4)	281.2 (304.3)	155.6 (196.5)	140.1 (206.4)	38.0 (39.8)	35.9 (49.6)
ln HF	5.02 (0.99)	5.21 (0.95)	4.52 (1.01)	4.38 (1.04)	3.23 (0.92)	2.86 (1.25)
LF/HF	6.0 (3.1)	4.7 (2.7)	8.4 (4.7)	8.8 (6.4)	7.4 (3.2)	6.6 (3.3)
ln LF/HF	1.66 (0.51)	1.42 (0.51)	1.99 (0.55)	1.99 (0.60)	1.91 (0.46)	1.75 (0.55)

**Table 3:** Crude and adjusted linear mixed models comparing activity types (3-levels), domains (2-levels) and their interaction for heart rate variability indices among blue-collar workers in the NOMAD cohort

Variables	Crude model			Adjusted model^a^		
	Estimate	95% CI	*p*	Estimate	95% CI	*p*
IBI (ms)						
Activity			< 0.01			< 0.01
Sitting	815.2	799.6 to 830.8		905.8	773.3 to 1038.3	
Standing	728.0	712.1 to 743.9		816.3	683.8 to 948.8	
Moving	583.0	566.2 to 599.8		674.0	541.2 to 806.8	
Domain						
Work	22.4	7.7 to 37.1	0.10	22.1	6.5 to 37.7	0.12
Interaction			< 0.01			< 0.01
Sitting	− 63.9	− 83.3		− 63.4	− 83.9	
Standing	− 23.3	− 43.3		− 22.8	− 43.9	
ln SDNN (ms)						
Activity			< 0.01			< 0.01
Sitting	3.95	3.88 to 4.00		5.15	4.77 to 5.53	
Standing	3.87	3.81 to 3.93		5.07	4.69 to 5.45	
Moving	3.63	3.57 to 3.70		4.83	4.45 to 5.22	
Domain			0.53			0.99
Work	− 0.04	− 0.09 to 0.02		− 0.02	− 0.07 to 0.04	
Interaction			0.30			0.66
Sitting at work	0.06	− 0.02 to 0.13		0.03	− 0.04 to 0.11	
Standing at work	0.03	− 0.05 to 0.10		0.01	− 0.07 to 0.09	
ln RMSSD (ms)						
Activity			< 0.01			< 0.01
Sitting	3.26	3.18 to 3.33		4.40	3.84 to 4.96	
Standing	2.97	2.89 to 3.04		4.10	3.54 to 4.66	
Moving	2.47	2.39 to 2.55		3.62	3.06 to 4.19	
Domain			0.22			0.19
Work	0.11	0.03 to 0.18		0.12	0.04 to 0.20	
Interaction			< 0.01			< 0.01
Sitting at work	− 0.16	− 0.26 to − 0.06		− 0.18	− 0.28 to − 0.07	
Standing at work	− 0.09	− 0.19 to 0.01		− 0.10	− 0.21 to 0.01	
ln LF (ms^2^/Hz)						
Activity			< 0.01			< 0.01
Sitting	6.37	6.22 to 6.52		9.88	8.93 to 10.83	
Standing	6.24	6.09 to 6.40		9.74	8.79 to 10.69	
Moving	4.61	4.44 to 4.78		8.16	7.21 to 9.12	
Domain			< 0.01			< 0.01
Work	0.39	0.23 to 0.56		0.39	0.22 to 0.55	
Interaction			0.06			0.09
Sitting at work	− 0.22	− 0.44 to − 0.01		− 0.21	− 0.42 to 0.00	
Standing at work	− 0.23	− 0.45 to − 0.02		− 0.21	− 0.43 to 0.00	
ln HF (ms^2^/Hz)						
Activity			< 0.01			< 0.01
Sitting	5.21	5.04 to 5.38		8.46	7.32 to 9.59	
Standing	4.40	4.22 to 4.57		7.62	6.47 to 8.75	
Moving	2.89	2.70 to 3.08		6.20	5.06 to 7.35	
Domain			0.20			0.22
Work	0.32	0.13 to 0.51		0.30	0.11 to 0.49	
Interaction			< 0.01			< 0.01
Sitting at work	− 0.51	− 0.76 to − 0.27		− 0.49	− 0.74 to − 0.25	
Standing at work	− 0.25	− 0.51 to 0.00		− 0.21	− 0.46 to 0.05	
ln LF/HF						
Activity			< 0.01			< 0.01
Sitting	1.42	1.33 to 1.51		1.65	0.86 to 2.44	
Standing	1.98	1.89 to 2.07		2.22	1.43 to 3.01	
Moving	1.76	1.66 to 1.86		1.97	1.18 to 2.76	
Domain			< 0.01			< 0.01
Work	0.12	0.03 to 0.24		0.13	0.03 to 0.23	
Interaction			< 0.01			< 0.01
Sitting at work	0.12	0.00 to 0.24		0.12	− 0.01 to 0.25	
Standing at work	− 0.09	− 0.21 to 0.04		− 0.10	− 0.24 to 0.03	

Data are presented as estimates of fixed effects, confidence intervals (95% CI) and *p* values. Leisure domain was regarded as reference

*NOMAD* New method for Objective Measurements of physical Activity in Daily Life, *RMSSD* square root of the mean squared differences of successive RR intervals, *SDNN* standard deviation of RR intervals, *LF* low frequency power, *HF* high frequency power, *LF/HF* low frequency power divided by high frequency

^a^Model adjusted for age, sex, smoking, body mass index, leisure time physical activity, job seniority, lifting and carrying, influence at work

The stratified analysis showed that for the non-smokers and for the high influence groups there were slight differences in the estimates and confidence intervals compared to the original results, but no changes in the statistical significance (results not shown). However, for smokers and low influence groups the interaction between activity and domain was no longer significant for RMSSD and HF power (Tables 4, 5). For the smokers group, the interaction between time and domain was also no longer significant for the sympathovagal balance (LF/HF).

**Table 4:** Crude and adjusted linear mixed models stratified by smoking status (smoke = yes) comparing activity types (3-levels), domains (2-levels) and their interaction for heart rate variability indices among blue-collar workers in the NOMAD cohort

Variables	Crude model			Adjusted model^a^		
	Estimate	95% CI	*p*	Estimate	95% CI	*p*
IBI (ms)						
Activity			< 0.01			< 0.01
Sitting	774.2	753.1 to 795.3		733.3	561.1 to 905.5	
Standing	682.5	660.6 to 704.3		640.3	468.0 to 812.7	
Moving	579.3	556.3 to 602.4		535.6	362.8 to 708.5	
Domain			0.68			0.75
Work	17.2	− 3.5 to 37.9		17.0	− 4.7 to 38.8	
Interaction			< 0.01			< 0.01
Sitting at work	− 43.8	− 70.9 to − 16.7		− 44.5	− 72.9 to − 16.1	
Standing at work	− 0.7	− 28.9 to 27.6		− 0.9	− 30.4 to 28.7	
ln SDNN (ms)						
Activity			< 0.01			< 0.01
Sitting	3.81	3.73 to 3.89		4.61	4.02 to 5.19	
Standing	3.72	3.63 to 3.81		4.51	3.92 to 5.09	
Moving	3.59	3.50 to 3.69		4.38	3.79 to 4.97	
Domain			0.36			0.40
Work	− 0.04	− 0.13 to 0.04		− 0.04	− 0.13 to 0.05	
Interaction			0.16			0.24
Sitting at work	0.10	− 0.01 to 0.22		0.10	− 0.02 to 0.22	
Standing at work	0.09	− 0.03 to 0.21		0.09	− 0.04 to 0.21	
ln RMSSD (ms)						
Activity			< 0.01			< 0.01
Sitting	3.11	2.99 to 3.22		3.96	3.06 to 4.86	
Standing	2.78	2.66 to 2.90		3.63	2.73 to 4.54	
Moving	2.47	2.35 to 2.60		3.34	2.43 to 4.25	
Domain			0.11			0.19
Work	0.09	− 0.03 to 0.22		0.08	− 0.05 to 0.21	
Interaction			0.25			0.30
Sitting at work	− 0.12	− 0.27 to 0.04		− 0.11	− 0.27 to 0.06	
Standing at work	− 0.01	− 0.17 to 0.16		0.01	− 0.17 to 0.18	
ln LF (ms^2^/Hz)						
Activity			< 0.01			< 0.01
Sitting	6.09	5.86 to 6.32		8.64	7.13 to 10.16	
Standing	5.80	5.57 to 6.04		8.35	6.84 to 9.87	
Moving	4.54	4.29 to 4.79		7.09	5.56 to 8.61	
Domain			< 0.01			< 0.01
Work	0.31	0.07 to 0.55		0.29	0.04 to 0.55	
Interaction			0.69			0.68
Sitting at work	− 0.08	− 0.39 to 0.24		− 0.07	− 0.41 to 0.26	
Standing at work	0.06	− 0.27 to 0.39		0.07	− 0.28 to 0.41	
ln HF (ms^2^/Hz)						
Activity			< 0.01			< 0.01
Sitting	4.86	4.60 to 5.12		6.93	5.22 to 8.64	
Standing	3.95	3.68 to 4.22		6.02	4.31 to 7.73	
Moving	2.84	2.55 to 3.13		4.95	3.22 to 6.67	
Domain			0.10			0.18
Work	0.25	− 0.04 to 0.55		0.22	− 0.09 to 0.53	
Interaction			0.06			0.08
Sitting at work	− 0.38	− 0.76 to 0.01		− 0.36	− 0.77 to 0.05	
Standing at work	0.01	− 0.39 to 0.41		0.04	− 0.38 to 0.47	
ln LF/HF						
Activity			< 0.01			< 0.01
Sitting	1.50	1.37 to 1.63		2.08	0.99 to 3.18	
Standing	2.00	1.86 to 2.14		2.58	1.49 to 3.67	
Moving	1.74	1.60 to 1.89		2.29	1.20 to 3.39	
Domain			< 0.01			< 0.01
Work	0.09	− 0.06 to 0.23		0.10	− 0.05 to 0.25	
Interaction			0.12			0.13
Sitting at work	0.13	− 0.06 to 0.32		0.12	− 0.08 to 0.31	
Standing at work	− 0.06	− 0.25 to 0.14		− 0.07	− 0.28 to 0.13	

Data are presented as estimates of fixed effects, confidence intervals (95% CI) and *p* values. Leisure domain was regarded as reference

*NOMAD* New method for Objective Measurements of physical Activity in Daily Life, *RMSSD* square root of the mean squared differences of successive RR intervals, *SDNN* standard deviation of RR intervals, *LF* low frequency power, *HF* high frequency power, *LF/HF* low frequency power divided by high frequency

^a^Model adjusted for age, sex, body mass index, leisure time physical activity, job seniority, lifting and carrying, influence at work

**Table 5:** Crude and adjusted linear mixed models stratified by influence at work (influence = low) comparing activity types (3-levels), domains (2-levels) and their interaction for heart rate variability indices among blue-collar workers in the NOMAD cohort

Variables	Crude model			Adjusted model^a^		
	Estimate	95% CI	*p*	Estimate	95% CI	*p*
IBI (ms)						
Activity			< 0.01			< 0.01
Sitting	821.3	792.7 to 849.9		1009.6	811.0 to 1208.2	
Standing	744.9	715.8 to 773.9		930.5	731.6 to 1129.3	
Moving	591.1	560.6 to 621.6		774.1	579.4 to 973.3	
Domain			< 0.01			0.01
Work	7.1	− 18.5 to 32.7		8.0	− 18.9 to 34.9	
Interaction			< 0.01			< 0.01
Sitting at work	− 52.6	− 85.8		− 55.2	− 89.8 to − 20.5	
Standing at work	− 22.4	− 56.4		− 23.0	− 58.4 to 12.5	
ln SDNN (ms)						
Activity			< 0.01			< 0.01
Sitting	3.90	3.80 to 4.00		5.41	4.84 to 5.98	
Standing	3.85	3.75 to 3.95		5.36	4.79 to 5.93	
Moving	3.66	3.55 to 3.77		5.17	4.60 to 5.75	
Domain			0.16			0.15
Work	− 0.12	− 0.22 to − 0.03		− 0.13	− 0.23 to − 0.03	
Interaction			0.02			0.04
Sitting at work	0.17	0.05 to 0.29		0.17	0.04 to 0.30	
Standing at work	0.09	− 0.03 to 0.22		0.10	− 0.04 to 0.23	
ln RMSSD (ms)						
Activity			< 0.01			< 0.01
Sitting	3.20	3.08 to 3.32		4.63	3.86 to 5.41	
Standing	2.97	2.85 to 3.09		4.40	3.63 to 5.18	
Moving	2.51	2.38 to 2.64		3.95	3.18 to 4.73	
Domain			0.79			0.72
Work	0.02	− 0.11 to 0.14		0.01	− 0.12 to 0.14	
Interaction			0.82			0.88
Sitting at work	− 0.05	− 0.21 to 0.11		− 0.04	− 0.22 to 0.13	
Standing at work	− 0.03	− 0.20 to 0.13		− 0.02	− 0.20 to 0.15	
ln LF (ms^2^/Hz)						
Activity			< 0.01			< 0.01
Sitting	6.24	5.97 to 6.51		10.07	8.62 to 11.51	
Standing	6.16	5.88 to 6.43		9.97	8.52 to 11.42	
Moving	4.52	4.23 to 4.81		8.35	6.90 to 9.81	
Domain			< 0.01			< 0.01
Work	0.32	0.05 to 0.59		0.30	0.02 to 0.58	
Interaction			0.57			0.63
Sitting at work	− 0.06	− 0.41 to 0.28		− 0.05	− 0.41 to 0.31	
Standing at work	− 0.19	− 0.54 to 0.17		− 0.17	− 0.54 to 0.20	
ln HF (ms^2^/Hz)						
Activity			< 0.01			< 0.01
Sitting	5.01	4.74 to 5.28		8.71	7.18 to 10.24	
Standing	4.32	4.05 to 4.60		8.01	6.48 to 9.55	
Moving	2.29	2.63 to 3.22		6.63	5.09 to 8.17	
Domain						
Work	0.15	− 0.14 to 0.44	0.75	0.13	− 0.17 to 0.44	0.85
Interaction			0.36			0.42
Sitting at work	− 0.27	− 0.64 to 0.11		− 0.25	− 0.65 to 0.14	
Standing at work	− 0.11	− 0.49 to 0.27		− 0.09	− 0.50 to 0.31	
ln LF/HF						
Activity			< 0.01			< 0.01
Sitting	1.47	1.32 to 1.62		1.54	0.41 to 2.67	
Standing	1.97	1.81 to 2.12		2.02	0.90 to 3.15	
Moving	1.65	1.49 to 1.82		1.71	0.58 to 2.84	
Domain			< 0.01			< 0.01
Work	0.17	0.02 to 0.32		0.17	0.01 to 0.32	
Interaction			0.04			0.04
Sitting at work	0.08	− 0.11 to 0.28		0.08	− 0.12 to 0.28	
Standing at work	− 0.15	− 0.35 to 0.05		− 0.16	− 0.36 to 0.05	

Data are presented as estimates of fixed effects, confidence intervals (95% CI) and p values. Leisure domain was regarded as reference

*NOMAD* New method for Objective Measurements of physical Activity in Daily Life, *RMSSD* square root of the mean squared differences of successive RR intervals, *SDNN* standard deviation of RR intervals, *LF* low frequency power, *HF* high frequency power, *LF/HF* low frequency power divided by high frequency

^a^Model adjusted for age, sex, smoke, body mass index, leisure time physical activity, job seniority, lifting and carrying

## Discussion

This study assessed heart rate variability (HRV) during sitting, standing and moving at work and leisure time in the NOMAD cohort. The results showed significant effects of domain and activity type on HRV indices. Generally, sympathetic modulation was higher at work than during leisure. Moving activity showed the lowest HRV indices, followed by standing still and sitting. The interaction between domain and activity type was also significant. That is, mean heart rate and parasympathetic modulation was lower for sitting and higher for moving during work, while no difference between work and leisure was found for standing. Sympathovagal balance was higher during work for sitting and moving, but showed no difference for standing.

Similarly to other studies, our findings indicate a significant effect of the activity types on HRV. Other studies have also shown the effect of body posture and physical activity on autonomic modulation (Pomeranz et al. 1985; Bernardi et al. 1996; Perini and Veicsteinas 2003; Rennie et al. 2003; Chan et al. 2007; Watanabe et al. 2007; Valentini and Parati 2009; Silva et al. 2015). Based on the above mentioned studies, it was expected that the highest HRV indices would be found while sitting, as a result of the vagal predominance during rest. On the other hand, we also expected a lower HRV during standing and moving, which can be attributed to vagal withdrawal and sympathetic predominance (Malliani et al. 1991; Michael et al. 2017).

Our findings also showed that the sympathetic modulation (LF) and sympathovagal balance (LF/HF) were higher during work, although LF can be influenced by both sympathetic and parasympathetic activity. These findings indicate that work has a differential effect on autonomic activity compared to leisure. Other studies have shown that increased sympathetic modulation is related to increased cardiovascular risk and mortality (Tsuji et al. 1994, 1996). Thus, increased sympathetic and reduced vagal activity at work can be part of the mechanism explaining why OPA has a negative effect on cardiovascular health.

It is already known that the relationship between physical activity and health depends on whether the activity occurs at work or leisure (Li et al. 2013; Holtermann et al. 2012a). Specifically, moderate and high levels of LTPA are associated with favorable health outcomes, while OPA shows no clear or even inverse relationship (Holtermann et al. 2012b, 2013; Allesøe et al. 2014; Saidj et al. 2014). Hallman et al. (2017) evaluated HRV during sleep and found beneficial effects of LTPA only when OPA was low. Thus, the autonomic cardiac modulation seems to be one possible physiological response behind the physical activity health paradox. However, the underlying mechanisms for a different autonomic regulation during the same physical activity type and body posture during work and leisure are unknown. One potential factor explaining this effect can be that the autonomy and mental load during performance of specific tasks differ between work and leisure, as shown in laboratory studies that simulated increased mental load (Hjortskov et al. 2004; Chandola et al. 2010). Thus, a stratified analysis was performed to verify if differences between work and leisure depend on influence at work. The findings suggest that influence at work modified the relationship between physical activity and the parasympathetic modulation of the heart. The interaction between activity and domain with regard to parasympathetic cardiac modulation (HF and RMSSD) was reduced and became statistically non-significant for the low influence group, but remained statistically significant for the high influence group. Since the high influence group presumably has the lowest stress levels, these results do not suggest that work stress can explain the moderating effect of domain on activity. However, as no data about mental stress was available we could not infer whether stress factors are responsible for the differences in parasympathetic activity between work and leisure during the same physical activity/posture.

For the smokers group, the interaction between activity and domain with regard to parasympathetic cardiac modulation (HF and RMSSD) was reduced and became statistically non-significant, but remained statistically significant for the non-smokers group. Thus, these results also suggest that smoking modified the relation between physical activity and the parasympathetic modulation of the heart.

### Strengths and limitations

The main limitations are lacking information about specific work tasks performed, and respiration rate which both could influence HRV. In addition, our findings on blue-collar workers may not be representative of the general working population, e.g. white-collar workers. Future studies could also include more recording days to allow some familiarization of the subjects with the devices and to remove potential bias, e.g. increased physical activity due to the use of the accelerometers. Information about diet, circadian clock, occupational activity and mental stress could also bring more insights about this issue. This is the first study, using objective measurements of physical activity and HRV for multiple days in a large and homogeneous socioeconomic sample, showing HRV differences between work and leisure during physical activities. This finding may contribute to the understanding of the health paradox of occupational and leisure-time physical activity.

## Conclusions

Differences in cardiac autonomic modulation between work and leisure domains were found, indicating a sympathetic predominance during work and parasympathetic predominance during leisure for sitting. Autonomic responses can be part of the mechanism that explains the differential effect of occupational and leisure time physical activity on health. Smoking and low influence at work modifies the relation between physical activity and the HRV.

## Abbreviations

ANOVA: Analysis of variance
BMI: Body mass index
HRV: Heart rate variability
HF: High frequency spectral power
IBI: Inter beat intervals
LF: Low frequency spectral power
LF/HF: Ratio between LF and HF
LTPA: Leisure time physical activity
NOMAD: New method for objective measurements of physical activity in daily life
OPA: Occupational physical activity
PNS: Parasympathetic nervous system
RMSSD: Square root of the mean squared differences of successive IBI
SDNN: Standard deviation of IBI
SNS: Sympathetic nervous system
